# City Mortality

**Published:** 1866-12

**Authors:** 


### CITY MORTALITY.

The following is the statement of the Health Officer of the city mortality for the month of November:

| | |
|---|---|
| Died in South Division, . . . | 137 |
| Died in West Division, . . . . | 172 |
| Died in North Division, . . . . | 73 |
| Total, . - . | 382 |
| Total in November, 1865, . . . | 299 |
| Total in October, 1866, . . . | 1170 |

*Ages.*—Under five years, 173; from five to ten, 20; from ten to twenty, 25; from twenty to thirty, 39; from thirty to forty, 36; from forty to fifty, 40; from fifty to sixty, 15; from sixty to seventy, 12; from seventy to eighty, 11; from eighty to ninety, 2; from ninety to one hundred, 1; unknown, 8. Total, 382.

*Nativities.*—Chicago, 162; United States, 72; Canada, 7; Denmark, 1; England, 5; France, 1; Germany, 51; Ireland, 39; Italy, 2; Norway, 15; Sweden, 1; Switzerland, 1; Scotland, 6; Newfoundland, 1; unknown, 18. Total, 382.

*Causes of Death.*—Accident, 16; Asthma, 3; Apoplexy, 1; Burned, 3; Cancer, 8; Childbirth, 4; Cholera, 12; Cholera Morbus, 4; Cholera Infantum, 1; Congestion of Brain, 6; Congestion of Lungs, 4; Consumption, 39; Convulsions, 47; Croup, 11; Cold, 1; Disease of Brain, 1; Disease of Heart, 2; Disease of Liver, 2; Disease of Kidney, 1; Disease of Hip, 1; Disease of Lungs, 2; Disease of Throat, 1; Delirium Tremens, 3; Decline, 3; Dropsy, 9; Diarrhœa, 5; Diphtheria, 12; Dysentery, 2; Drowned, 2; Erysipelas, 3; Intermittent Fever, 8; Scarlet Fever, 6; Spotted Fever, 1; Typhoid Fever, 18; Fever not stated, 4; Gun-shot wound, 3; Hemorrhage, 3; Hydrocephalus, 3; Inflammation of Brain, 9; Inflammation of Bowels, 4; Inflammation of Lungs, 13; Inflammation of Liver, 1; Intemperance, 4; Measles, 1; Marasmus, 1; Old Age, 12, Phthisis Pulmonalis, 2; Paralysis, 3; Still-born, 8; Summer Complaint, 5; Scald, 1; Teething, 1; Tuberculosis, 2; Diabetes, 1; Ulcer, 1; Whooping Cough, 17; Wound, 1; unknown, 36. Total, 382.

---

As we close the twenty-third volume of the Journal, our thanks are due to the numerous friends who have assisted us by their subscriptions and by their good wishes. A few names on our list of subscribers are still disfigured by the black cross of unpaid debt, but the majority have testified their appreciation of our efforts by a prompt settlement of dues. As no physician in the Northwest can afford to be without the Journal, and as we do not propose to retain the names of delinquents upon our subscription lists, we hope that these few tardy debtors will no longer copy the example of that class of their patients who do not hesitate to receive, without rendering compensation or thanks for, professional services.

---

WANTED, the July and August Nos. of the Journal, for 1865.

---

**MARRIED.**—In this city, Dec. 4th, at the residence of J. W. Sykes, Esq., by Prof. S. C. Bartlett, D. D., BENJAMIN DURHAM, M. D., to Miss LUCY M. CLARK, both of Chicago.

